# Convenient screening of the reproductive toxicity of favipiravir and antiviral drugs in *Caenorhabditis elegans*

**DOI:** 10.1016/j.heliyon.2024.e35331

**Published:** 2024-07-26

**Authors:** Kimiyasu Shiraki, Mizuki Mishima, Noriaki Sato, Yasuo Imoto, Kiyoji Nishiwaki

**Affiliations:** a Senri Kinran University, Suita, Osaka, 565 0873, Japan; b Department of Bioscience, School of Science and Technology, Kwansei Gakuin University, 2-1 Gakuen, Sanda, 669-1339, Japan; c Division of Health Medical Intelligence, Human Genome Center, The Institute of Medical Science, The University of Tokyo, Tokyo, 108-8639, Japan; d Japan Textile Products Quality and Technology Center, 5-7-3 Shimoyamatedori, Chuo-ku, Kobe, 650-0011, Japan

**Keywords:** Antivirals, Favipiravir, Ribavirin, Reproductive toxicity, Caenorhabditis elegans, Arrested embryos, Telomerase reverse transcriptase, RNA dependent RNA polymerase

## Abstract

Reproductive toxicity is one of the major concerns in drug development. Thus, we have developed its screening system using *Caenorhabditis elegans,* which has a life cycle of three days and similar coding genes as humans. Antiviral nucleoside analogs used for acute infections are known to cause reproductive toxicity, contraindicated for pregnant women, and are used for comparing their reproductive toxicity in *C. elegans* and experimental animals. None of the drug treatments affected the number of offspring and the concentrations without toxicity to nematodes were consistent with no cytotoxicity or toxicity in experimental animals or humans. Favipiravir, ribavirin, molnupiravir (NHC), acyclovir, ganciclovir, zidovudine, and thalidomide significantly increased the incidence of arrested embryos but amenamevir, letermovir, and guanosine did not. RNA-dependent RNA polymerase (RdRp) inhibitors, in the order of favipiravir, ribavirin, and NHC increased the incidence of arrested embryos, possibly due to the specificity of favipiravir for RdRp and less cytotoxicity. RdRp inhibitors would impair RNA interference through RdRp expressed by telomerase reverse transcriptase during embryogenesis and cause embryo-fetal toxicity. The incidence of arrested embryos may be affected by differences in the substrate specificity of DNA polymerases and metabolism between *C. elegans*, animals, and humans. The concordance between the results of the screening system for reproductive toxicity of antivirals in *C. elegans* and those in experimental animals based on the International Council for Harmonisation of Technical Requirements for Pharmaceuticals for Human Use, reproductive toxicology confirms its appropriateness as a screening system for reproductive toxicity. Favipiravir and zidovudine were the least toxic to *C.elegans* among the antiviral drugs examined.

## 1 Introduction

The coronavirus disease-2019 (COVID-19) pandemic has brought attention to anti-RNA-dependent RNA polymerase (RdRp) drugs as a therapeutic drug for COVID-19. In particular, favipiravir, ribavirin, and molnupiravir, a prodrug of β-d-N4-hydroxycytidine (NHC, EIDD-1931), have attracted attention. These RdRp inhibitors showed reproductive toxicity in experimental animals and are contraindicated for pregnant women [[1], [2], [3]].

In drug development, the effects of drugs on early embryogenesis, organogenesis, and spermatogenesis are tested in reproductive toxicity evaluation. The International Council for Harmonisation of Technical Requirements for Pharmaceuticals for Human Use (ICH) defines reproductive toxicology as a method of measuring reproductive toxicity in animals in S5, “Guidelines for the Detection of Reproductive Toxicity in Pharmaceuticals for Human Use” of the Safety Guidelines [4]. Testing the toxic effects of drugs on early embryogenesis involves examining the mortality of embryo/fetus after 0–7 days of gestation, and determining the toxic effects of drugs on organogenesis involves examining the mortality of embryo/fetus and anomaly of the fetus after 7–17 days of gestation. In humans, evaluation of the toxic effect on sperm requires 2–3 months as that is the time required for sperm maturation from the testis and subsequent ejaculation.

*Caenorhabditis (C.) elegans* reproduces 300 progenies per hermaphrodite adult via self-fertilization with a life cycle of three days, and the genomes of both humans and *C. elegans* encode 20000–25000 and 19000 coding genes, respectively [5,6]. Because of the similarity of the genomes and gene functions, *C. elegans* has become an important animal model in various fields, including toxicology [[7], [8], [9], [10], [11]]. Despite the limitations of *C. elegans* toxicity testing, *C. elegans* toxicity has been correlated with toxicity in mice and rats [10].

The offspring of *C. elegans* is formed within the body of a nematode through the processes of fertilization by sperm and development of fertilized eggs in a hermaphrodite nematode, with 1320 and 3118 reproduction-related genes in humans and *C. elegans,* respectively [12]. Although sperm maturation requires 2–3 months after receiving the drug in humans, it is completed in a day in *C. elegans*. Accordingly, *C. elegans* may be a suitable alternative model for evaluating the effects of drugs on the reproductive systems of experimental animals and humans.

Oral antiviral nucleoside analogs show reproductive toxicity in experimental animals compared with the other drug development models, and we selected them for this study. The concordance between the results of the screening system for reproductive toxicity of oral antivirals in *C. elegans* and those in experimental animals confirms its appropriateness as a screening system for reproductive toxicity. Although it is not clear why RdRp inhibitors exhibit reproductive toxicity, RdRp activity is found to be expressed in early embryogenesis. Telomerase reverse transcriptase (TERT) expressed in early embryogenesis possesses both reverse transcriptase and RdRp activity, and RNA synthesized by TERT-RdRp induces RNA interference to regulate transcription [13,14]. We hypothesized that nucleoside RdRp inhibitors would inhibit RNA synthesis by TERT-RdRp, impair post-translational regulation by injured RNA interference, and cause embryo death during embryogenesis [15].

In this study, we have reproduced the reproductive toxicity of thalidomide and nine oral antivirals obtained in laboratory animals in the *C. elegans* system, indicating that drugs can be screened for reproductive toxicity in *C. elegans* and experimental animals.

## 2 Materials and methods

### 2.1 Arrested embryos born from an adult hatched and grew from a single embryo in the presence of an antiviral drug

The nematode used in this study, the Bristol strain designated N2 of *C. elegans,* was grown and maintained on nematode growth (NG) agar and handled using the method described by Brenner [16]. We selected oral antiviral drugs for this study as they have shown the most frequent reproductive toxicity in experimental animals compared with those of other drug delivery models. The antiviral drugs used were favipiravir, an RdRp inhibitor (Toyama Chemical Co. Ltd., Toyama, Japan); ribavirin, an RdRp inhibitor (Wako Pure Chemical Industries, Osaka, Japan); NHC, an RdRp inhibitor (Sigma-Aldrich, Darmstadt, Germany); acyclovir, a herpesvirus DNA synthesis inhibitor (Wako Pure Chemical Industries, Osaka, Japan); ganciclovir, a cytomegalovirus DNA synthesis inhibitor (Tanabe Seiyaku Co., Ltd. Osaka, Japan); zidovudine, a reverse transcriptase inhibitor; thalidomide, an antineoplastics immunomodulatory agent (Tokyo Chemical Industry Co., Ltd., Tokyo, Japan); amenamevir, a helicase-primase inhibitor of herpes simplex virus (HSV) and varicella-zoster virus (VZV) (Astellas Pharma, Inc. Tokyo, Japan); and letermovir, a cytomegalovirus DNA terminase complex inhibitor (Selleck Co. Osaka, Japan).

*C. elegans* is hermaphroditic, and its life cycle is as follows: laid eggs hatch in about half a day, larvae develop, and larvae become adults in two and a half days at 20 °C. Adults produce about 300 fertilized eggs. Ten nematode embryos were set on three 10-cm plates grown on *Escherichia coli* from nematode culture plates. After 2 days, the spawned nematode embryos were transferred to plates containing no drug or the drug containing various concentrations.

The embryos were placed on 20 agar plates with no drug, 3.5 μg/mL of amenamevir, 20 μg/mL of letermovir, 0.5 μg/mL of zidovudine, 2 μg/mL of thalidomide, 50 or 100 μg/mL of favipiravir, 3 or 6 μg/mL of acyclovir, ganciclovir, and NHC, an active form of molnupiravir, or 3.6 and 7.2 μg/mL of ribavirin. Favipiravir, acyclovir, ganciclovir, and ribavirin are the guanosine analogs, and guanosine was used at 6 μg/mL as a natural control substance. Antiviral drug concentrations, including the maximum drug concentration (C_max_) values, were obtained from their package inserts [2,3,[17], [18], [19], [20], [21], [22], [23], [24]]. The half-maximal cytotoxic concentrations (CC_50_) of favipiravir, acyclovir, ganciclovir, NHC, ribavirin, letermovir, amenamevir, and zidovudine were >1,000, >4,500, 237 to 380, 2.5 to 98.7, >1,000, >286, >14.5, and >133.6 μg/mL respectively [3,[25], [26], [27], [28], [29], [30], [31], [32]].

Nematode embryos were placed, one per dish, in 20 60-mm petri dishes of NG agar medium with or without an antiviral drug and incubated at 20 °C. When the nematodes started laying embryos, most hatched within 10–12 h [33]; they were removed within 48 h and incubated for 24 h for hatching and prevention of contamination. The dishes were fixed in 5 % formalin (Wako Pure Chemical Corporation, Osaka, Japan), and the nematodes and arrested embryos in an agar plate were counted under a dissecting microscope. Each experiment on the effects of antiviral drugs on nematode embryos was repeated with a control (no drug) and only two or three drugs.

### 2.2 Classification of arrested embryos into stages including the bean stage, comma stage, and 1.5-fold stage

**Fig. 1:**
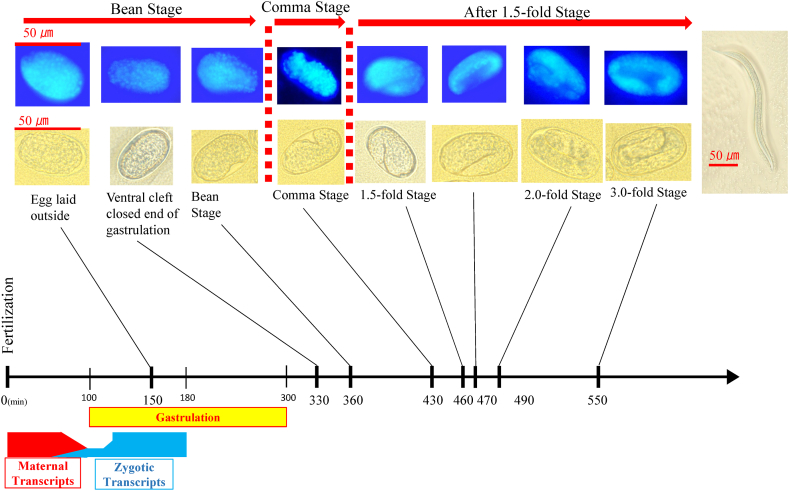
Photographs show plain and DAPI-stained embryos in the order of embryonic stages of development [34]. Gastrulation is an early developmental process in the vertebrate body's plan for organogenesis [35,36]. Red and blue curves indicate the maternal-to-zygotic transition from maternal transcripts and transcripts from the zygotic genome [37]. A morphological classification of arrested embryos between the gastrula and bean stages would be more suitable for the purposes of this study. The gastrulation stage corresponds to implantation. Maternal and zygotic transcripts may be a source of miRNAs for RNA interference by TERT-RdRp in post-translational regulation from gametogenesis to embryogenesis. However, the distinction between the gastrula and bean stages is difficult to observe in actuality, as shown in the figure. As it is easier and more definite to distinguish, the arrested embryos were classified as the bean, comma, and 1.5-fold and later stages. Arrested embryos up to the bean, comma, and 1.5-fold and later stages may roughly correspond to the embryogenesis, fetal resorptions/fetal resorptions before organogenesis, and lethal teratology/external malformation, respectively, as reproductive toxicity in experimental animals. The red arrows indicate the boundary among the bean, comma, and 1.5-fold stages.

The time of developmental arrest was determined by observing the morphology of the embryos, either unstained or stained with 4′,6-diamidino-2-phenylindole (DAPI), under a fluorescent microscope (Nikon Solutions Co., Ltd., Tokyo, Japan). About 10 images with shifting focus of the arrested embryos to determine their stage were captured and the arrested embryos were classified into three groups: the bean stage, comma stage, and 1.5-fold stage, respectively (Fig. 1).

### 2.3 Statistical analysis

Differences in the incidence of arrested embryos in the antiviral treatments were assessed using the Student t-test and one-way repeated-measures analysis of variance (ANOVA), followed by the Bonferroni/Dunn method or the Dunnett method (Statcel). A comparison of the ratio of the bean, comma, and 1.5-fold stages in arrested embryos after antiviral treatment was performed using chi-squared tests. Here, the comma and 1.5-fold stages were grouped, and the comparison between the control and each antiviral treatment was made with subsequent Bonferroni correction of p-values. The correlation between the incidence of arrested embryos and the ratio of arrested embryos was analyzed by using Pearson's product-moment correlation. Statistical significance was set at P < 0.05.

## 3 Results

### 3.1 Effect of oral antiviral drugs on the incidence of arrested embryos

There was no significant difference in the number of total offspring (fertilized nematodes and arrested embryos) per nematode among treatments without and with guanosine, amenamevir, letermovir, zidovudine, thalidomide, favipiravir, acyclovir, ganciclovir, ribavirin, and NHC in each set of the experiment (data not shown). The concentrations of all antiviral drugs had no apparent effects on nematode size, shape, motility, and the shape of the arrested embryo under the binocular microscope.

**Fig. 2:**
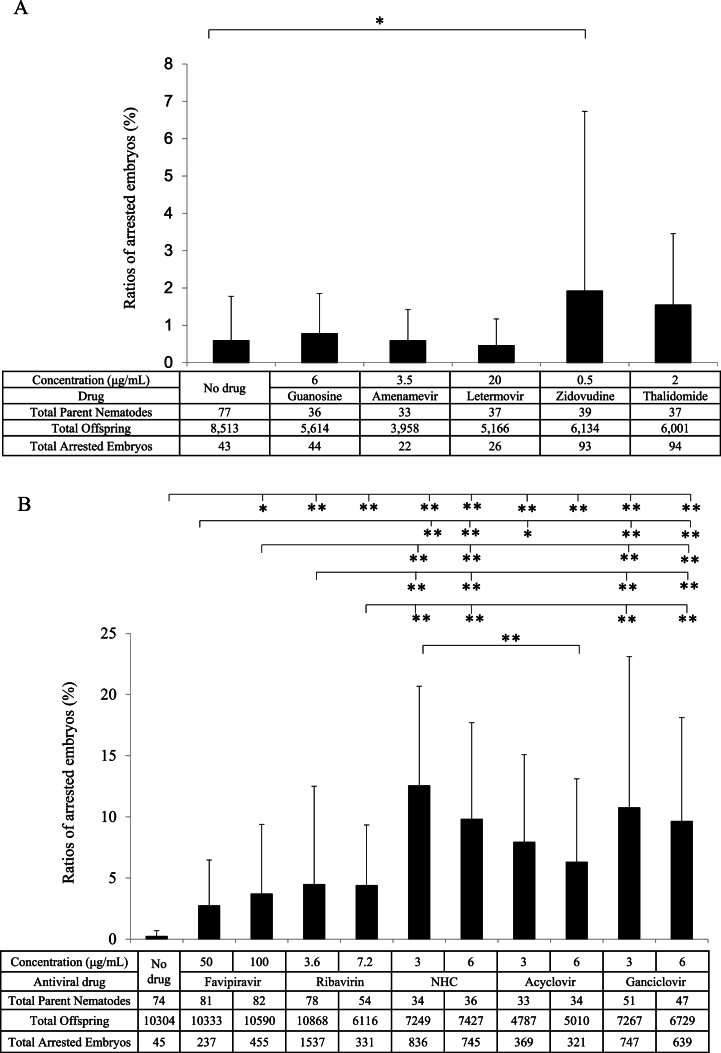
Comparison of incidences of arrested embryos treated with drugs. A and B show the comparative incidences (%) of arrested embryos of *Caenorhabditis elegans* treated with guanosine, amenamevir, letermovir, zidovudine, thalidomide, favipiravir, ribavirin, NHC, acyclovir and ganciclovir, respectively. The tables show antiviral concentrations and total numbers of parent nematodes, offspring, and arrested embryos. The column and error bars show the mean incidence of arrested embryos deduced from the arrested embryos from one parent nematode in the table and its standard deviation. The comparison of incidence among treatment groups was analyzed by one-way analysis of variance followed by the Bonferroni-Dunn method. * and ** indicate P < 0.05 and P < 0.01, respectively. NHC is the active compound of molnupiravir.

Fig. 2A shows the incidence of arrested embryos about the total number of parent nematodes, offspring, and arrested embryos among *C. elegans* cultures treated without and with guanosine, amenamevir, letermovir, zidovudine, and thalidomide. Amenamevir and letermovir did not increase the incidence of arrested embryos compared with no drug control and guanosine treatment. Although the number of arrested embryos by zidovudine and thalidomide varied considerably among the nematodes with greater deviations, the incidence of arrested embryos was higher in the zidovudine and thalidomide treatments than in the control treatment, according to the Student t-test (P < 0.05 and P < 0.01, respectively). Guanosine, which is not toxic for reproduction, did not increase the frequency of arrested embryos, nor did the antiviral drugs, amenamevir and letermovir, which showed no reproductive toxicity in experimental animals. Zidovudine and thalidomide, which showed reproductive toxicity in experimental animals, increased the incidence of arrested embryos.

Fig. 2B shows the incidence of arrested embryos in relation to the total number of parent nematodes, offspring, and arrested embryos in the series of experiments. Drug treatments with favipiravir, ribavirin, NHC, acyclovir, and ganciclovir significantly increased the incidence of arrested embryos compared with that of no drug treatment, as shown by using the Dunnett method (P < 0.01). The incidences between drug treatment groups were compared using a one-way ANOVA followed by the Bonferroni/Dunn method (Fig. 2B). The incidence of arrested embryos with favipiravir treatment was lower than those with most other drugs. The incidences of ganciclovir and NHC treatments were higher than those of the other treatments.

### 3.2 Effects of oral antiviral drugs on the developmental stages of arrested embryos

**Fig. 3:**
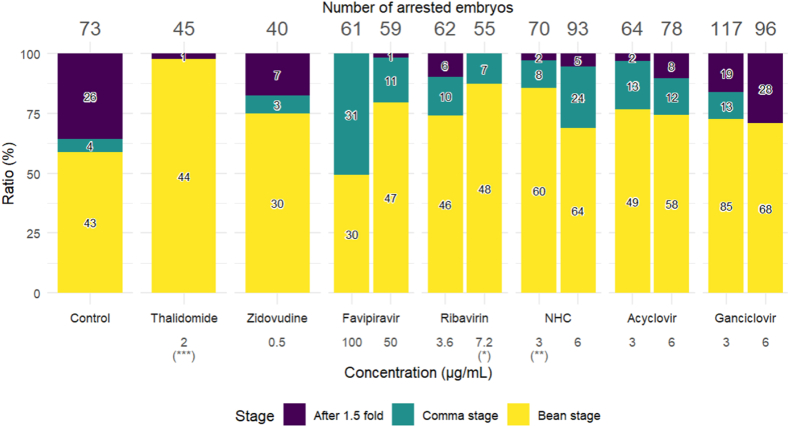
Comparison of drug effects on the ratios of arrested stages of embryos treated with antivirals. Figure shows the comparative incidence (%) of arrested embryos before the bean stage, comma stage, and after the 1.5-fold stage in antiviral drug treatments of nematodes. X-axis shows the antiviral treatments and their concentrations, and Y-axis shows the incidence. The raw numbers of the arrested embryos in antiviral drug treatments of nematodes are shown in the bars, and the sum of the numbers is depicted at the top of each bar. The chi-square tests were used to compare the ratios of the control with the antiviral treatments. *, ** and *** indicated P < 0.05, P < 0.01 and P < 0.001, respectively. NHC is the active compound of molnupiravir.

We compared the proportion of the bean, comma, and 1.5-fold stages between the control and the antiviral treatments. thalidomide, ribavirin, and NHC treatments showed statistically significant differences compared to the control group, but no significant differences were observed in the other drug treatments (Fig. 3). Thalidomide increased the bean stage-arrested embryos but not the 1.5-fold stage-arrested embryos; this assay is based on arrested embryos, indicating non-lethal teratology would not be reflected here as is that observed in humans. Ribavirin and NHC increased the proportion of the bean stage-arrested embryos. The percentages of the bean stage-arrested embryos ranged from 49 to 98 %, depending on the drugs and their concentrations. The proportions of three stage groups were specific to each drug and not due to the mechanism of the drug actions of nucleoside antiherpetic drugs and RdRp inhibitors causing developmental impairment from fertilization to hatching.

**Fig. 4:**
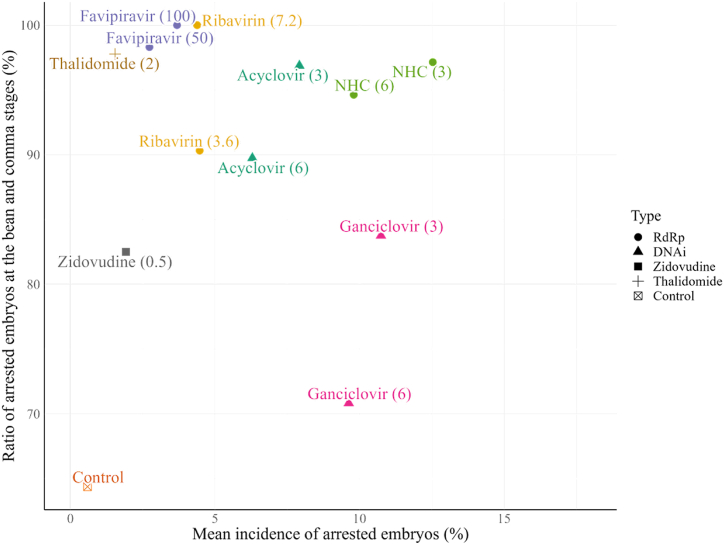
Correlation between the incidence of arrested embryos and the ratio of arrested embryos treated with antivirals at the bean and comma stages. Although the ratio of arrested embryos of the bean and comma stages clustered around 70–90 %, favipiravir and ribavirin belong to the lower arrested embryo incidence group, while ganciclovir and NHC belong to the higher arrested embryo incidence group. There was no statistically significant correlation between the incidence of arrested embryos and the ratio of arrested embryos (Pearson's product-moment correlation = 0.085, P = 0.784). Drug concentrations (μg/mL) are shown in parentheses next to the drug name. NHC is the active compound of molnupiravir. ●: RdRp inhibitors, Favipiravir, Ribavirin, and NHC. ▲: DNA polymerase inhibitors, Acyclovir and Ganciclovir.

Fig. 4 shows the relationship between the incidence of arrested embryos and the percentage of the bean and comma stage-arrested embryos. There was no statistically significant correlation between the incidence of arrested embryos and the percentage of stage-arrested embryos (Pearson's product-moment correlation = 0.085, P = 0.784). Favipiravir and ribavirin showed lower incidences of arrested embryos than those of the other antiviral agents, whereas ganciclovir and NHC showed higher incidences. The relationship between the incidence of arrested embryos and the ratio of arrested embryos up to the comma stage indicated the drug-specific mechanism of developmental impairment from fertilization to hatching.

## 4 Discussion

The oral antiviral drugs, including RdRp inhibitors, were examined for reproductive toxicity in *C. elegans,* and the results were consistent with those in previous experimental animals. Although RdRp has been thought to be absent in mammals, including humans, TERT possesses both reverse transcriptase and RdRp activity, and RNA synthesized by TERT-RdRp induces RNA interference to regulate transcription [13,14]. Telomeres, which are located at the ends of chromosomes and consist of repeating sequences, shorten by 50–150 bases with each cell division, and the gradual loss of telomere sequences terminates cell division, a process known as senescence [38,39]. Furthermore, TERT elongates shortened telomere length during development from fertilized eggs to early embryos (up to implantation) to recover shortened telomere length [40,41], and TERT-RdRp produces microRNAs (miRNAs) as post-translational regulators from gametogenesis to embryogenesis [13,[42], [43], [44]]. The timing of inhibition of embryogenesis in experimental animals by RdRp inhibitors coincides with the timing of expression of TERT-RdRp, indicating it is a candidate for lethal impairment of RNA interference by RdRp inhibitors. Lomibuvir**,** a hepatitis C virus non-nucleoside RdRp inhibitor, inhibits RNA synthesis by the RdRp activity of TERT-RdRp but does not affect DNA synthesis by the TERT activity of TERT-RdRp [15]. We hypothesized that nucleoside RdRp inhibitors would inhibit RNA synthesis by TERT-RdRp, impair post-translational regulation by injured RNA interference, and cause embryo death during embryogenesis.

Amenamevir is a helicase-primase inhibitor used for treating HSV and VZV infections. Helicase-primase is essential for separating double strands into two single strands and creates a replication fork during DNA synthesis [22,[45], [46], [47]]. Amenamevir did not show reproductive toxicity in mice and rabbits at concentrations used in humans [23].

Letermovir is a cytomegalovirus DNA terminase complex inhibitor for prophylaxis of cytomegalovirus infection and disease; fertility, early embryonic development, and male reproductive system toxicities were not observed in letermovir-treated experimental animals [21].

Zidovudine is a nucleoside analog RT inhibitor used for the prevention and treatment of human immunodeficiency virus-1 (HIV-1) infection. Although zidovudine did not show teratogenicity in the rats and rabbits, it caused an increase in the incidence of fetal resorptions in both animals, and zidovudine exposure reduced dose-dependent blastocyst formation in fertilized mouse oocytes in vitro [48]. Although rats showed an increase in early resorptions and a decrease in litter size, no other evidence of developmental toxicity was noted in rats or rabbits [49]. Zidovudine may inhibit TERT-mediated elongation of telomeres but does not impair RNA interference as postulated as the action of RdRp inhibitors, because it is not a substrate of RdRp. Zidovudine is used for the prevention of maternal-fetal HIV-1 transmission after 14 weeks of gestation, avoiding the first 10 weeks of gestation as the potential teratogenic period.

Thalidomide was developed as a sedative; when it was used to treat women in the early stages of pregnancy, it caused malformations in the limbs, ears, and other parts of the fetus. Thalidomide shows its various pharmacological actions in angiogenesis, immunomodulation, and anti-tumor actions in multiple myeloma; the mechanism of teratogenicity of thalidomide has been elucidated as the inhibition of cereblon, and its associated ubiquitin ligase activity and inhibition of angiogenesis by inhibiting basic fibroblast growth factor [[50], [51], [52], [53], [54]]. Thalidomide increased the proportion of arrested embryos before the bean stage, and this may correspond to the increase in the number of absorbed embryos and the decrease in the number of litters in experimental animals [24,[55], [56], [57]]. It is unclear why thalidomide causes developmental arrest before the comma stage, but it may be related to the fact that thalidomide suppresses GC-rich hTERT core promoter-reporter gene expression [58]. Thalidomide did not arrest embryo development later than the comma stage nor cause abnormal mobility and deformity in the nematode. Therefore, teratogenicity of thalidomide observed in human fetuses would not be observed in nematodes. Reproductive toxicity screening using C. elegans does not appear suitable for non-lethal teratogenicity.

Acyclovir induces teratogenicity in whole embryo culture systems and pregnant animals [[59], [60], [61]]. However, no higher risk estimate than the general population and no pattern of defects were observed in the following registry systems: the Acyclovir Pregnancy Registry with birth defects, the Lamotrigine Pregnancy Registry with birth defects, the Sumatriptan Pregnancy Registry with birth defects, and the Valacyclovir and Bupropion Pregnancy Registry [62]. In population-based historical cohort studies of 837,795 cases in Denmark, acyclovir and valacyclovir did not increase major birth defects [63].

Ganciclovir significantly increased the incidence of arrested embryos compared with that of no drug treatment and showed a tendency for a higher incidence of arrested embryos later than the bean stage of the embryo compared to other drugs. Ganciclovir induces teratogenicity and impairs spermatogenesis in experimental animals [20,64]. A liver transplant recipient treated with oral ganciclovir during the first trimester showed no evidence of teratogenicity [65].

Ribavirin has a broad spectrum of anti-RNA viral activities against parainfluenza virus, respiratory syncytial virus, and chronic hepatitis C [[66], [67], [68]]. Ribavirin causes limb, rib, eye, and central nervous system teratogenicity and fetal death in pregnant hamsters [69] and impairment of rat and human spermatogenesis [[70], [71], [72]]. Ribavirin inhibits both RNA and DNA synthesis and has an increased risk of teratogenicity comparable to that of cytosine arabinoside [73]. The Ribavirin Pregnancy Registry prospectively collected data on 464 pregnant women exposed to ribavirin, and preliminary results did not indicate clear signals of human teratogenicity [74].

Favipiravir shows a broad-spectrum of life-threatening anti-RNA viral activities against influenza, severe fever with thrombocytopenia syndrome, COVID-19, rabies, and Ebola virus infections in animals and humans [[75], [76], [77], [78], [79], [80], [81], [82], [83], [84], [85], [86]].

In experimental animals, favipiravir exhibits teratogenicity and embryotoxicity, and its toxicity in comparison with that of ribavirin and valacyclovir is shown in Table 1 [17,19,77]. As shown in Table 1, the toxicity of favipiravir and antiviral drugs in early rat embryogenesis has been evaluated by pre-implantation loss rate, number of surviving fetuses and premature deaths, the loss rate of post-implantation, and rate of fetuses with abnormalities and their embryo-fetal developmental toxicity by the number of live fetuses, post-implantation loss, and rate of fetuses with abnormalities. Favipiravir and ribavirin increased post-implantation mortality and reduced the number of living embryos/fetuses compared with valacyclovir. Although ribavirin showed fetal anomalies at lower rat/human AUC ratios during embryo-fetal development, toxicity of favipiravir and ribavirin was observed more in early embryogenesis. The proportion of favipiravir- and ribavirin-arrested embryos at the bean and comma stages were high, as shown in Fig. 3. We hypothesized that favipiravir and ribavirin would impair transcriptional regulation by RNA interference by TERT-RdRp, utilizing the maternal and zygotic transcripts shown in Fig. 1 at the early embryogenesis stage; favipiravir and ribavirin inhibited embryogenesis in experimental animals and *C. elegan*s. Compared to valacyclovir, favipiravir and ribavirin were more toxic during early embryogenesis than during embryo-fetal development in experimental animals as shown in Table 1.

**Table 1:** Summary of toxicity of favipiravir and antiviral drugs during early rat embryogenesis.

Group	Dose (mg/kg/day)	Rat/Human AUC ratio^$^	Mortality of embryo/fetus				Anomaly of fetus
			Mean pre-implantation loss rate (%)	Mean number of surviving fetuses	Mean number of premature deaths	Mean post-implantation loss rate (%)	Rate of fetuses with abnormalities (%)
							External malformation
Vehicle control	0	N/A	13.8	11.8	1.1	8.4	0
Favipiravir	10	0.12	4.0	13.6	0.6	4.3	0
	30	0.37	5.7	6.8*	7.9**	55.5**	0
Ribavirin	30	0.14	10.0	8.8	4.4*	36.1*	14.7*
	100	0.41	7.2	0.6**	12.6**	95.0**	62.5
Valacyclovir	100	1.1	9.7	13.4	0.3	2.1	0
	200	2.3	6.9	14.5	0.8	5.1	0.9
	400	4.9	5.9	9.4	6.1*	39.3*	2.0

Administration period: 0–7 days gestation (until implantation).

Summary on the 20th day of gestation to observe the endometrium and foetation.

$, Human AUC was based on the following dose: favipiravir, 1600 mg × 2/day (loading dose for influenza patients); ribavirin, 400 mg × 2/day (dose for 60–80 kg patients); valacyclovir, 1000 mg × 3/day (dose for zoster patients).

Statistically significant difference from the vehicle control: *, p < 0.05; **, p < 0.01.

The table is provided with permission by Fujifilm Toyama Chemical Co., Ltd [17].

The authors obtained permission from Pharmacology & Therapeutics to reuse this table [77].

Favipiravir has been considered highly toxic, but it is a potent anti-RNA virus drug and could prevent infection even in the cases of a needle stick from a patient infected with the Ebola virus [83]. Among 17508 COVID-19-infected patients treated with favipiravir, 2934 had hyperuricemia, and 1205 had abnormal liver function [87]. There was no significant difference in the incidence of liver function abnormalities in the favipiravir-treated and control groups [85,[88], [89], [90]]. Favipiravir treatment did not result in human sperm disorders in clinical trials unlike ribavirin treatment, and testicular toxicity was not observed in monkeys at 6.3 and 1.2 times the maximum human exposure to favipiravir for two and six weeks, respectively [17,19,77].

As the embryo-fetotoxicity of favipiravir has been observed in experimental animals, its administration to pregnant women is contraindicated; unfortunately, its use in pregnant women during the COVID-19 epidemic has been reported [91,92]. Of the 29 pregnancies exposed to favipiravir, five were electively terminated, and 24 delivered live births within the normal range of weight, length, and head circumference without major congenital malformation. Three infants were born premature, and one child had a patent foramen ovale. The authors indicated that favipiravir was unlikely to be a major human teratogen [91]. In addition, nine cases of exposure to favipiravir during pregnancy have been reported: one spontaneous abortion, two elective terminations, and five full-term live deliveries. One preterm live delivery patient died on the fifth day. Physiological jaundice and transient respiratory distress were recorded in the two-term infants. None of the infants had congenital malformations, and the authors indicated that favipiravir was unlikely to be a major teratogen [92].

Molnupiravir (NHC) is a cytosine ribonucleoside analog, and its triphosphate form inhibits SARS-CoV-2 RdRp activity. The diphosphate form of NHC is the substrate of ribonucleotide reductase and is converted to the deoxyribose form [46,93]. The 2ʹ-deoxyribose form of NHC is incorporated into the host cell DNA as a cytidine analog, causing mutagenesis in CHO–K1 cells [93]. Oral administration of molnupiravir to pregnant rats during organogenesis resulted in embryonic lethality and teratogenicity and was contraindicated for pregnancy [3].

The toxicity of acyclovir, ganciclovir, and ribavirin in experimental animals has not directly been observed in humans. Human DNA polymerase substrate specificity and species-specific metabolisms may be more stringent than those in nematodes and rats. The lower incidence of favipiravir-arrested embryos compared to those of ribavirin-, NHC-, or other anti-herpes drug-arrested embryos was assumed to be due to the absence of impaired DNA synthesis, as favipiravir has a high specificity for RdRp inhibition without cellular DNA synthesis, which is expected from the CC_50_ value. Thus, in this study, we established a convenient and reliable screening system for reproductive toxicity using *C. elegans,* and this system can conveniently be applied to select candidate compounds for drug development.

## 5 Conclusion

We developed a convenient and useful screening system for reproductive toxicity using *C. elegans* before experimental animals. When reproductive toxicity is found, contraindications for pregnant women are important to avoid the risk of reproductive toxicity. The concordance between the results of the screening system for reproductive toxicity of antivirals and thalidomide in *C. elegans* and those in experimental animals confirms its appropriateness as a screening system for reproductive toxicity.

## Funding statement

No funding was obtained for the purpose of this work.

## Data availability statement

The data supporting the findings of this study are available from the corresponding author upon reasonable request.

## CRediT authorship contribution statement

**Kimiyasu Shiraki:** Writing – review & editing, Writing – original draft, Visualization, Validation, Supervision, Software, Resources, Project administration, Methodology, Investigation, Funding acquisition, Formal analysis, Data curation, Conceptualization. **Mizuki Mishima:** Writing – review & editing, Resources, Methodology, Investigation, Formal analysis, Data curation, Conceptualization. **Noriaki Sato:** Writing – review & editing, Writing – original draft, Visualization, Validation, Software, Methodology, Formal analysis, Data curation, Conceptualization. **Yasuo Imoto:** Writing – review & editing, Visualization, Supervision, Methodology, Formal analysis, Conceptualization. **Kiyoji Nishiwaki:** Writing – review & editing, Writing – original draft, Supervision, Methodology, Data curation, Conceptualization.

## Declaration of competing interest

The authors declare that they have no known competing financial interests or personal relationships that could have appeared to influence the work reported in this paper.
